# Inhibition of Polymicrobial Biofilms of *Candida albicans*–*Staphylococcus aureus*/*Streptococcus mutans* by Fucoidan–Gold Nanoparticles

**DOI:** 10.3390/md21020123

**Published:** 2023-02-13

**Authors:** Nazia Tabassum, Fazlurrahman Khan, Min-Gyun Kang, Du-Min Jo, Kyung-Jin Cho, Young-Mog Kim

**Affiliations:** 1 Marine Integrated Biomedical Technology Center, The National Key Research Institutes in Universities, Pukyong National University, Busan 48513, Republic of Korea; 2 Research Center for Marine Integrated Bionics Technology, Pukyong National University, Busan 48513, Republic of Korea; 3 Department of Food Science and Technology, Pukyong National University, Busan 48513, Republic of Korea

**Keywords:** anti-biofilm, marine-derived, mono-species, inhibition, polymicrobial biofilms, Fu–AuNPs

## Abstract

The polymicrobial proliferation and development of complex biofilm morphologies by bacterial and fungal pathogens in the host are some of the key factors contributing to the failure of antimicrobial treatments. The polymicrobial interaction of *Candida albicans* and some bacterial species has been extensively studied in both in vitro and in vivo model systems. Alternative strategies for disrupting polymicrobial interaction and biofilm formation are constantly needed. Among several alternative strategies, the use of nanoparticles synthesized using a natural product in the treatment of microbial infection has been considered a promising approach. The current study aimed to synthesize gold nanoparticles (AuNPs) using a natural product, fucoidan, and to test their efficacy against mono and duo combinations of fungal (*Candida albicans*) and bacterial (*Staphylococcus aureus*/*Streptococcus mutans*) biofilms. Several methods were used to characterize and study Fu–AuNPs, including UV-vis absorption spectroscopy, FTIR, FE-TEM, EDS, DLS, zeta potential, and XRD. The concentration-dependent inhibition of early-stage biofilms and the eradication of mature biofilms of single species of *C. albicans*, *S. aureus*, and *S. mutans* have been observed. Early biofilms of a dual-species combination of *C. albicans* and *S. aureus*/*S. mutans* were also suppressed at an increasing concentration of Fu–AuNPs. Furthermore, Fu–AuNPs significantly eradicated the established mature biofilm of mixed species. The treatment method proposed in this study, which involves the use of marine-bioinspired nanoparticles, is a promising and biocompatible agent for preventing the growth of polymicrobial biofilms of bacterial and fungal pathogens.

## 1. Introduction

Antimicrobial resistance (AMR), caused by a diverse range of microbial pathogens such as bacteria, viruses, and fungi, is a worldwide concern [1,2]. Increasing AMR in microbial pathogens is a huge burden on the global economy, requiring vast sums of money to treat patients [2,3]. The majority of antimicrobial efficacy has been evaluated by determining the minimum inhibitory concentration (MIC) in planktonic mono-species microbial cell cultures using Clinical and Laboratory Standards Institute (CLSI) guidelines [4]. However, when these screened antimicrobial agents were applied in in vivo systems under physiological conditions, they did not show efficacy at the determined MIC values [5,6].

The failure (i.e., decreased efficacy) of in vivo antimicrobial agent application systems has been reported due to the availability of polymicrobial interaction of microbial species in the host [7]. Polymicrobial interactions can occur at the interspecies and cross-kingdom levels [8,9]. The interaction of multiple microbes and biofilm progression in a variety of host mouths, skin, lungs, gastrointestinal tract, and vulvovaginal tissues has been extensively researched [10,11,12]. A growing number of reports describe the formation of polymicrobial biofilms between *Candida* spp., particularly *C. albicans*, and various bacterial species [11,13,14]. Polymicrobial biofilm formation has been linked to a variety of human diseases, including oral cavity infection, otitis media, chronic lung infection, burn wounds, urinary tract infection, and medical device-related infection [15,16]. Several studies show that polymicrobial interactions at the interspecies or cross-kingdom level result in increased tolerance to antimicrobial agents, host immune responses, and a variety of other environmental stress conditions [17].

As a result, antimicrobial treatment failure against biofilm-forming microbial pathogens is becoming more common, necessitating the development of alternative polymicrobial disease-fighting strategies. Nanomaterials are increasingly being used to treat biofilms [18,19,20], due to their different mechanisms compared to antibiotics or antifungals [21]. Nanoparticles, particularly metallic ones, have been widely used in the treatment of microbial pathogens due to several advantages, including specific targeting, an effective drug delivery system, less cytotoxicity, and controlled release [22,23,24]. A growing number of research studies has focused on using naturally occurring substances from plants, algae, fungi, bacteria, or animals in the synthesis of metallic nanoparticles [25,26]. The use of marine-derived materials in the synthesis of nanoparticles has received particular attention due to their ease of availability, low cost in the isolation of active molecules, biocompatibility, biodegradability, and potential bioactive properties [27,28,29,30].

The current study sought to synthesize gold nanoparticles using fucoidan, a marine-derived long-chained sulfated polysaccharide derived from brown algae, marine seaweeds, and invertebrates, such as sea urchins and sea cucumbers [31]. Fucoidan has been isolated from a variety of brown algae, including *Undaria pinnatifida*, *Cladosiphon okamuranus*, *Laminaria saccharina*, and *Fucus vesiculosus* [32,33]. The fucoidan polymer is structurally made up of (1→3)-linked or (1→4)-linked L-fucose residues with sulfate groups at the C-2 and C-4 positions, and a branch point formed by the attachment of fucose or another sugar [33]. It is a source of therapeutic and medicinal compounds, with anti-viral, anti-oxidant, anti-inflammatory, anti-coagulant, anti-thrombotic, anti-aging, gut, and digestive health applications [34,35,36]. 

Bacterial pathogens, such as *Staphylococcus aureus* and *Streptococcus mutans,* and fungal pathogens such as *C. albicans* were used in the current study as reference microbial pathogens to test the biofilm inhibition action of Fu–AuNPs. The molecular mechanism underlying the formation of mixed biofilms by *C. albicans*, *S. aureus*, and *S. mutans* has been well documented, and these microbes have been co-isolated from human tissues and organs [11]. AuNPs have been reported to have a non-cytotoxic effect, to be highly stable and biocompatible, and to be used effectively as a delivery system [28,37,38]. The synthesized Fu–AuNPs demonstrated efficacy against bacterial (*S. aureus* and *S. mutans*) and fungal pathogens (*C. albicans*). Furthermore, newly synthesized Fu–AuNPs effectively inhibited initial-stage biofilms and dispersed mature biofilms of mono- and dual-species.

## 2. Results

### 2.1. Synthesis and Characterization of Fu–AuNPs

**Figure 1:**
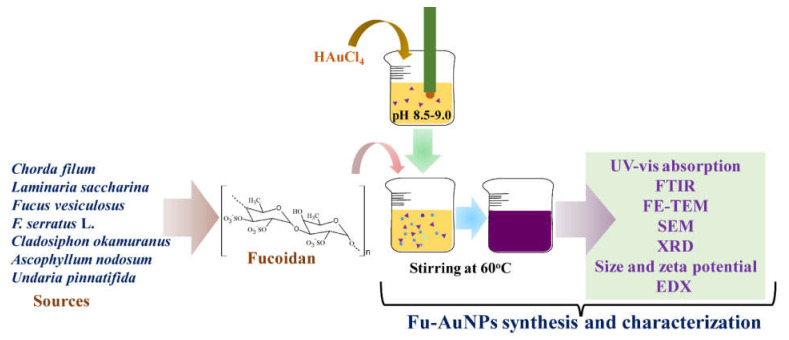
A representation of the detailed process of the synthesis and characterization of Fu–AuNPs.

During the in situ synthesis, the color of the chloroauric acid solution gradually changed from yellow to deep, wine-red with the addition of fucoidan, indicating the synthesis of the AuNPs. The diagrammatic illustration of the synthesis of Fu–AuNPs using fucoidan is depicted in Figure 1.

**Figure 2:**
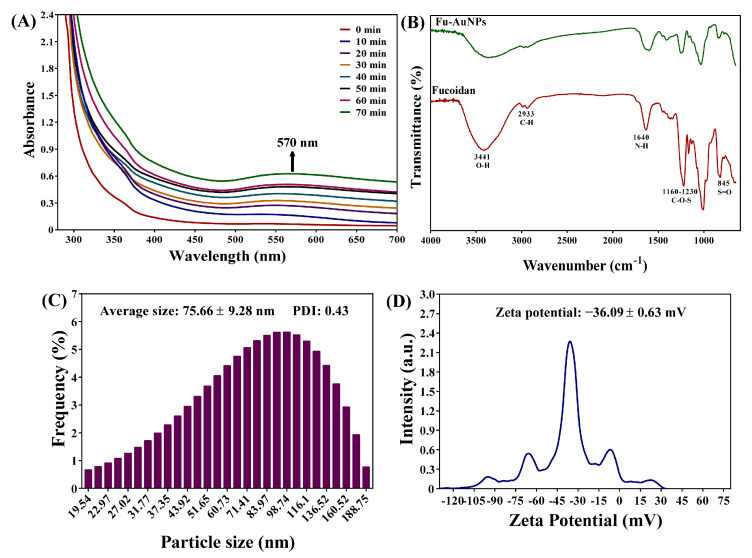
(**A**) UV-visible absorption spectra, (**B**) FTIR spectra of fucoidan and Fu–AuNPs, (**C**) Distribution of Fu–AuNP particle size, and (**D**) Zeta potential of Fu–AuNPs.

In addition to in situ monitoring, UV-visible absorption spectroscopy of NPs was performed during the chemical reaction time. The absorption spectra increased continuously, with a maximum absorption peak at 570 nm (Figure 2A). The wine-red color and the gradual increase in the absorption spectra during the chemical reaction proved the preliminary synthesis of AuNPs. The FTIR analysis is another instrumental-based characterization of NPs synthesis, and the results revealed that Fu–AuNPs have some vibration band characteristics. Fucoidan vibration band characteristics were discovered in FTIR spectra at 845 cm^−1^ (S=O) and 1160-1230 cm^−1^ (C-O-S) (Figure 2B). Similar bands existed in Fu–AuNP spectra. The hydroxyl group (O-H) and C-H stretching in Fu–AuNPs and fucoidan were observed at 3441 cm^−1^ and 2933 cm^−1^, respectively (Figure 2B). The N-H bending of amines is represented by the characteristic band at 1640 cm^−1^.

**Figure 3:**
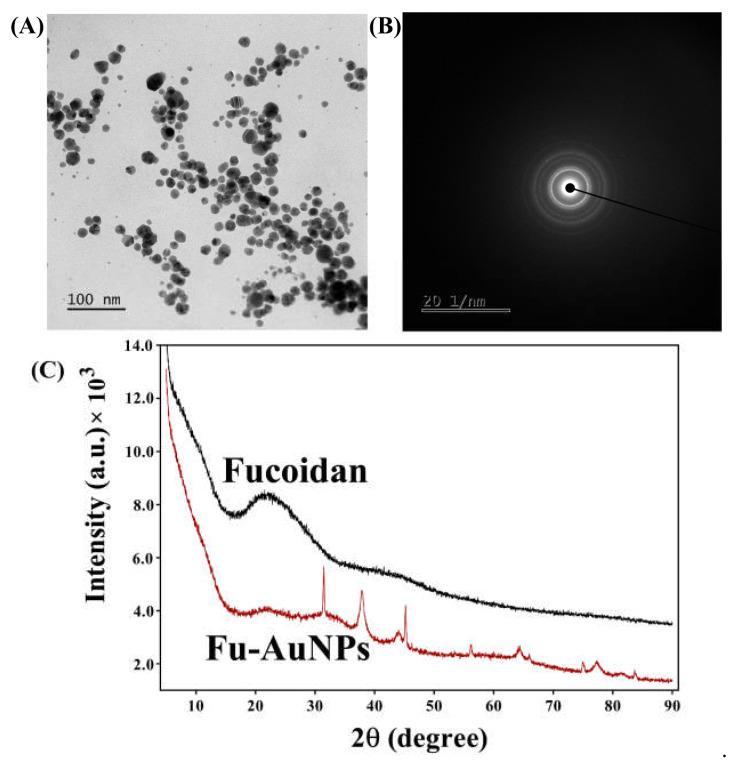
Instrumental characterization of Fu–AuNPs. (**A**) FE-TEM image of Fu–AuNPs, (**B**) SAED of Fu–AuNPs, and (**C**) XRD spectra of Fu–AuNPs and fucoidan.

**Figure 4:**
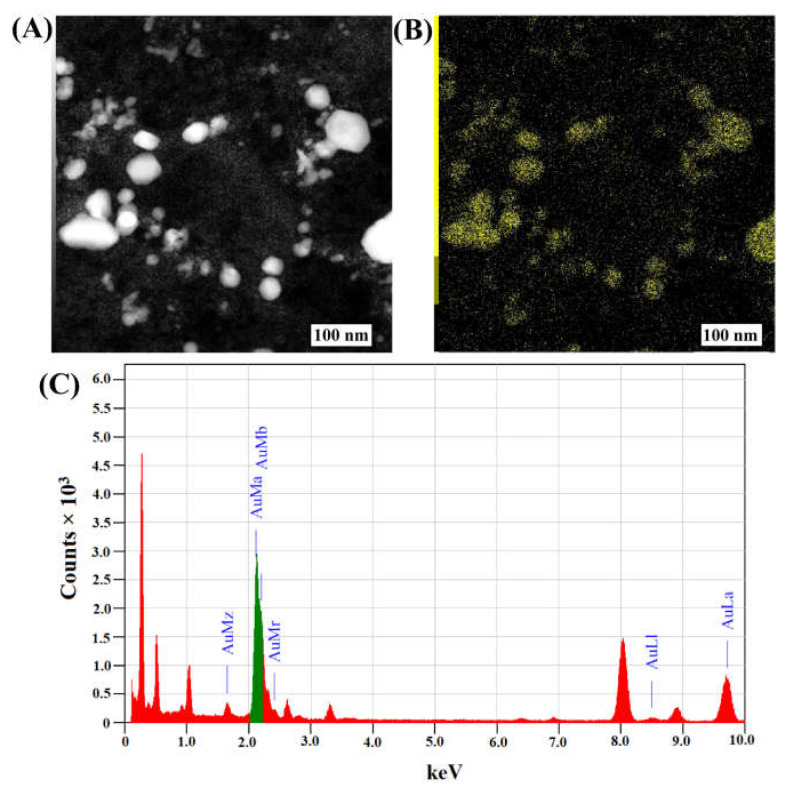
Elemental mapping and XRD analysis. (**A**) SEM image of Fu–AuNPs, (**B**) Mapping of Au element, and (**C**) EDS spectra of Fu–AuNPs.

The average size of the Fu–AuNPs was determined to be 75.66 ± 9.28 nm (Figure 2C). The zeta potential of Fu–AuNPs was determined to be −36.09 ± 0.63 mV (Figure 2D). The morphology of the Fu–AuNPs examined using FE-TEM revealed the presence of both spherical and nonspherical shapes (Figure 3A). The scanning electron microscopy image also shows the spherical and nonspherical shape of the Fu–AuNPs (Figure 4A). The presence of Debye-Scherrer rings in SAED indicates their crystalline nature, as determined by FE-TEM imaging of the Fu–AuNPs (Figure 3B). The crystallinity of the Fu–AuNPs was also tested using XRD spectra. In the XRD spectra, some of the characteristic peaks at 2ϴ were found to be 31.4°, 37.8°, 45.1°, 56°, 64.3°, and 77.3°, which are similar to the unique characteristics peaks found in the case of several green-synthesized AuNPs (Figure 3C). The energy-dispersive X-ray analysis of the Fu–AuNPs revealed the presence of a distinct spectrum between 16 and 32° (Figure 3C). The elemental mapping of Fu–AuNPs demonstrated the presence of Au metal (Figure 4B), and the elemental composition of the EDS revealed an abundance of the Au element (Figure 4C).

### 2.2. MIC and MBC, or MFC, Values of Fu–AuNPs towards Microbial Pathogens 

The microbroth dilution method was used to determine the antibacterial and antifungal properties of Fu–AuNPs. The MIC value of Fu–AuNPs was determined by observing microbial growth in a microtiter plate and measuring the OD_600_ of microbial cell growth. The MIC values for bacterial and *C. albicans* were discovered to be different (Table 1). The MIC values of Fu–AuNPs towards *S. aureus* and *C. albicans* planktonic cells were the same, 1024 µg/mL. However, for *S. mutans,* the MIC value was >512 µg/mL. There were no visible bacterial colonies of *S. mutans* at 1024 μg/mL, indicating that the MIC value of Fu–AuNPs against *S. mutans* is between 512 and 1024 μg/mL. Similarly, no visible colonies of *S. aureus* or *C. albicans* were found at a concentration of 1024 μg/mL Fu–AuNPs, which was considered the MBC (minimum bactericidal concentration) and MFC (minimum fungicidal concentration) value. It is clear that Fu–AuNPs had a one-fold higher MBC or MFC value against *S. aureus*, *S. mutans*, and *C. albicans* as compared to the MIC values (Table 1). Additionally, the MBC or MFC/MIC ratio in all cases was equal to two, indicating that Fu–AuNPs have bactericidal and fungicidal effects on various microbial pathogens.

### 2.3. Inhibitory Effect of Fu–AuNPs towards Single- and Mixed-Species Biofilms

**Figure 5:**
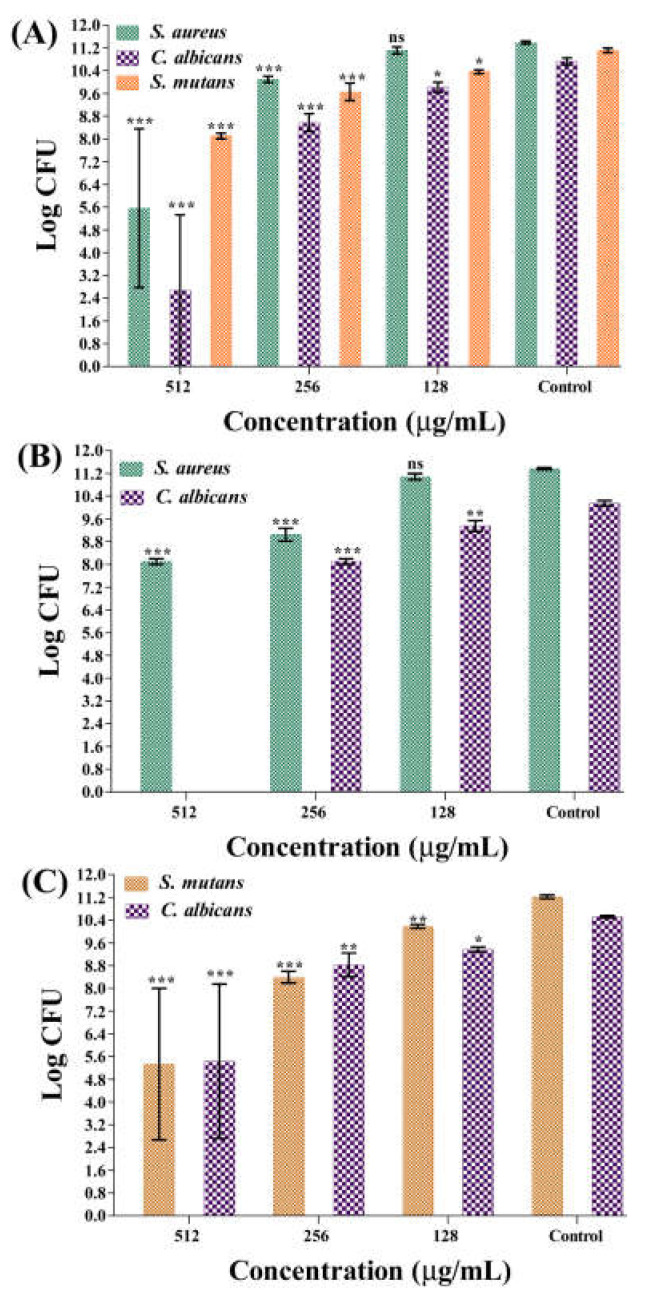
Biofilm inhibitory effect of Fu–AuNPs towards single- and mixed-species biofilms. (**A**) log CFU of *S. aureus*, *S. mutans*, and *C. albicans* cells from single-species biofilms, (**B**) log CFU of *C. albicans* and *S. aureus* cells from *C. albicans + S. aureus* biofilms, and (**C**) log CFU of *C. albicans* and *S. mutans* cells from *C. albicans* + *S. mutans* biofilms. *** *p* < 0.0001, ** *p* < 0.01, and * *p* < 0.05 indicate statistical significance, whereas ns is non-significance.

The initial stage of biofilm inhibition of single and polymicrobial species was tested using a subinhibitory concentration of Fu–AuNPs. The biofilm inhibitory effect was estimated using the plate-counting method. The inhibitory effect on mono-species biofilms was concentration-dependent for *S. aureus*, *S. mutans*, and *C. albicans* biofilms (Figure 5A). The maximum inhibition of a single-species biofilm was discovered to occur at 512 µg/mL concentration. At 512 µg/mL concentrations, the CFU values of *S. aureus*, *S. mutans*, and *C. albicans* were found to be 5.8, 3.0, and 8.0 log reductions, respectively. Similarly, the concentration-dependent inhibition of co-biofilms formed by the concurrent growth of *C. albicans* with *S. aureus* and *S. mutans* cultures was noted (Figure 5B,C). The biofilm of *C. albicans* was completely inhibited at 512 µg/mL of Fu–AuNPs, whereas 3.2 log CFU of *S. aureus* cells from the mixed biofilm of *C. albicans + S. aureus* was inhibited (Figure 5B). Similarly, at 512 µg/mL concentration, the maximum biofilm inhibition of the mixed biofilm of *C. albicans* + *S. mutans* was determined. At this concentration, *S. mutans* and *C. albicans* from the mixed biofilm were inhibited to 5.8 and 5.0 log CFU, respectively (Figure 5C).

### 2.4. Microscopic Examination of Single- and Mixed-Species Biofilms Treated with Fu–AuNPs 

**Figure 6:**
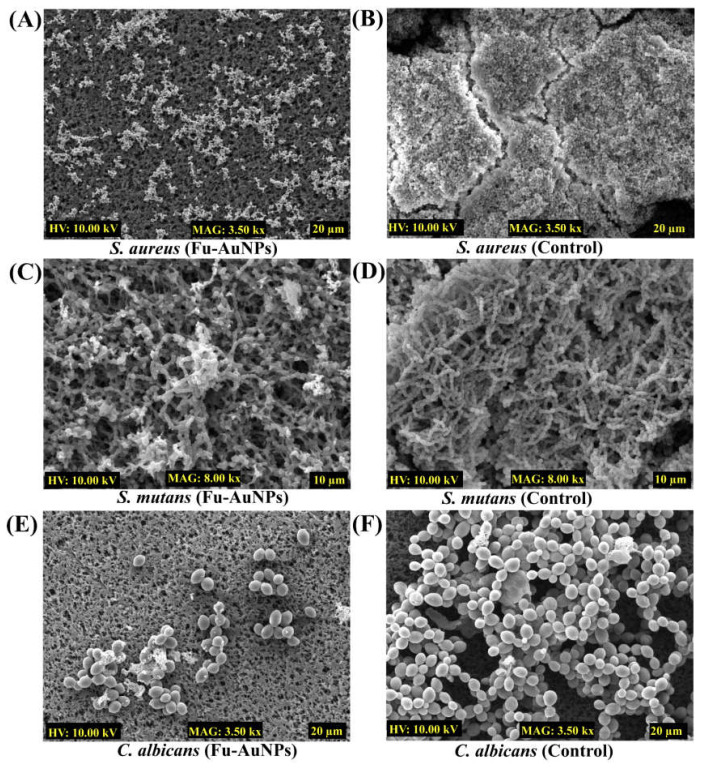
Mono-species biofilm architecture treated with Fu–AuNPs. (**A**) *S. aureus* biofilms treated with Fu–AuNPs, (**B**) Control biofilm of *S. aureus*, (**C**) *S. mutans* biofilm treated with Fu–AuNPs, (**D**) Control biofilm of *S. mutans*, (**E**) *C. albicans* biofilms treated with Fu–AuNPs, (**F**) Control biofilms of *C. albicans*.

**Figure 7:**
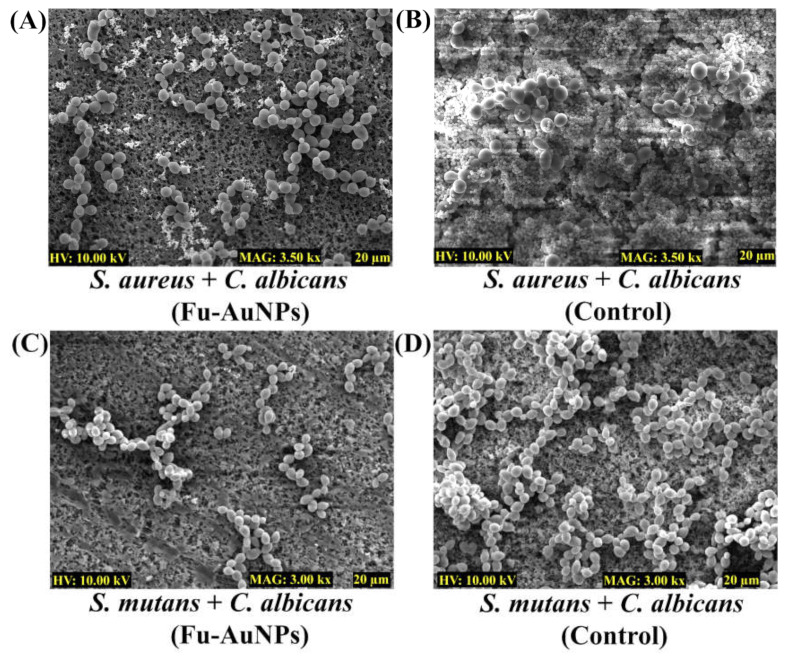
Mixed biofilm architecture treated with Fu–AuNPs. (**A**) *S. aureus* + *C. albicans* biofilms treated with Fu–AuNPs, (**B**) Control group of *S. aureus* + *C. albicans* biofilms, (**C**) *S. mutans* + *C. albicans* biofilms treated with Fu–AuNPs, and (**D**) Control biofilms of *S. mutans + C. albicans*.

The inhibitory consequence of Fu–AuNPs on the architecture of both single- and mixed-species biofilms was investigated by scanning electron microscopy (SEM). The SEM image of the untreated *S. aureus*, *S. mutans*, and *C. albicans* depicted that the biofilm layer that had aggregated on the nylon surface was highly dense (Figure 6B,D,F). Fu-AuNP--treated *S. aureus*, *S. mutans*, and *C. albicans* biofilms resulted in low populations of cells on the membrane’s surface (Figure 6A,C,E). The presence of less-adhered biofilm cells in comparison to untreated biofilm cells suggests that the single-species biofilm was inhibited at the initial stage of formation when treated with Fu–AuNPs. A similar effect was observed with biofilms of *C. albicans* mixed with *S. aureus*/*S. mutans* and treated with Fu–AuNPs (Figure 7). 

Similarly, when the mixed-species biofilms of *C. albicans* + *S. aureus*/*S. mutans,* treated with Fu–AuNPs, were examined using SEM, a similar effect was observed (Figure 7). *C. albicans* mixed biofilms treated with Fu–AuNPs, either with *S. aureus* (Figure 7A) or with *S. mutans* (Figure 7C), showed an inhibition of cell adherence on the membrane’s surface compared to the control group, which had a very thick mixed population of cells (Figure 7B,D). According to the SEM analysis, Fu–AuNPs have inhibitory effects on the biofilm formation of mono-species and polymicrobial biofilms of various bacterial species, including *C. albicans*. 

### 2.5. Dispersal of Established Mature Biofilms of Single or Mixed Species by Fu–AuNPs 

**Figure 8:**
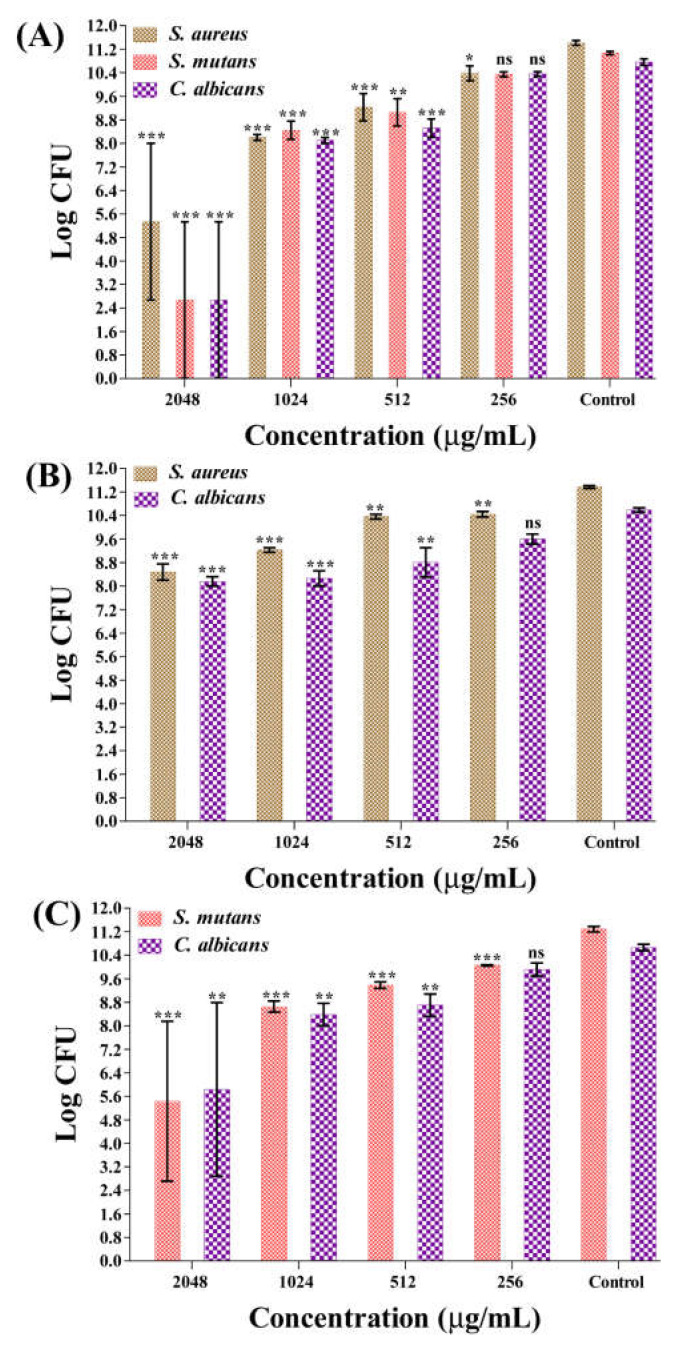
Dispersal effect of Fu–AuNPs towards mature biofilms of single or mixed microbial cells. (**A**) log CFU of *S. aureus*, *S. mutans*, and *C. albicans* cells from a mature, single-species biofilm, (**B**) log CFU of *S. aureus* and *C. albicans* cells from *C. albicans* + *S. aureus* mature, mixed biofilms, and (**C**) log CFU of *S. mutans* and *C. albicans* cells from *C. albicans* + *S. mutans* mature, mixed biofilms. *** *p* < 0.0001, ** *p* < 0.01, and * *p* < 0.05 indicate statistical significance, whereas ns is non-significance.

The disinfection efficacy of Fu–AuNPs against established mature biofilms was also investigated by treating mature single-species biofilms of *C. albicans*, *S. aureus*, *S. mutans*, and mixed-species biofilms of *C. albicans* combined with *S. aureus*/*S. mutans,* with different concentrations of Fu–AuNPs. Three concentrations of Fu–AuNPs, including >MIC, MIC, and sub-MIC values, were tested to study the complete eradication of both mono- and polymicrobial mature biofilms. Although a concentration-dependent eradication of mono-species and mixed biofilm mature biofilms was discovered, the eradication effect varied (Figure 8A–C). The dispersal effect of single- and mixed-species mature biofilms was found to be more effective when Fu–AuNPs were treated with > MIC values. The maximum eradication of mature single-species biofilms, comprising *S. aureus*, *S. mutans*, and *C. albicans*, was found to be greater at >MIC (2048 µg/mL) values of 6.0, 8.4, 8.0 log CFU, respectively (Figure 8A). The eradication of the mature mixed biofilm of *S. aureus*+*C. albicans* was found to be maximal at the >MIC value (2048 µg/mL), with efficacy values of 2.9 log CFU for *S. aureus* and 2.4 log CFU for *C. albicans* (Figure 8B). Similarly, the maximum eradication of the mature mixed biofilm of *C. albicans* + *S. mutans* by Fu–AuNPs at sub-MIC values (2048 µg/mL) was found to occur at log CFU values of 5.8 and 4.8, respectively, against *S. mutans* and *C. albicans* (Figure 8C). Based on the findings, it is concluded that the eradication of mixed-species biofilms was slightly lower than the eradication of single-species biofilms.

## 3. Discussion

The pathogenic microorganism coexisted with non-pathogenic microbes in the host and maintained its ecological balance by promoting mutual growth, metabolic signaling, and protection from the host immune system [39,40,41]. There are several tissues and organs in the host where polymicrobial diseases have been reported [10,42]. One of the failures in antimicrobial treatment has been identified as polymicrobial interaction [7]. The polymicrobial interaction causes robust biofilm formation in the host as well as on the surface of medical devices, which become a source of infection [10]. The increased biofilm formation caused by the symbiotic interaction of microbial species in the host leads to increased antimicrobial resistance [43]. The polymicrobial interaction of *C. albicans* with bacterial pathogens, such as *S. aureus*, *S. mutans*, *S. gordonii*, and *S. oralis,* is a well-studied example of polymicrobial interaction [11,44]. These bacterial pathogens and *C. albicans* have frequently been isolated from different host tissues and organs together [45]. Thus, increasing the mortality rate of a polymicrobial disease patient is always encouraged, in order to develop alternative strategies to combat polymicrobial infections [10,46]. Fucoidan, a marine product, was used as a reducing agent in the green synthesis of gold nanoparticles, and is capable of controlling the formation of mixed biofilms by *S. aureus* and *S. mutans* in collaboration with *C. albicans*. Although previous research reported that Fu–AuNPs inhibited single-species biofilms, primarily *Pseudomonas aeruginosa* biofilms, polymicrobial biofilms have not been reported [47]. 

The appearance of a wine-red color of the reaction mixture and specific absorption spectra (570 nm) in the UV-vis absorption spectroscopy confirmed the successful synthesis of the Fu–AuNPs, as seen previously in several reports [47,48]. The FTIR spectra show that some characteristic bands are unique to AuNPs and fucoidan, as previously identified in fucoidan-derived AuNPs and fucoidan [47,49]. The spherical shape of the Fu–AuNPs is similar to that of the previously synthesized fucoidan–gold nanoparticles [47,50]. Furthermore, several other gold nanoparticles synthesized using the natural product were discovered to be spherical, as well as non-spherical, in shape [47,48,51]. The size variation of nanoparticles synthesized using a natural product has been frequently reported. The size of the synthesized Fu–AuNPs was 75.66 ± 9.28 nm, which is in the range of previously synthesized fucoidan-mediated gold nanoparticles [50]. The synthesized Fu–AuNPs were found to be highly stable, as evidenced by the high zeta potential (−36.09 ± 0.63 mV), which is consistent with previous reports [50,52].

The physiochemically characterized Fu–AuNPs were used to test the inhibition of single- and mixed-species biofilms containing bacterial and *C. albicans* pathogens. The MIC values against bacteria and *C. albicans* were determined prior to the biofilm assays. The results showed that there is a one-fold higher MIC (1024 µg/mL) value against *S. aureus* and *C. albicans*, compared to the previously reported MIC (512 µg/mL) value against *Pseudomonas aeruginosa* [47]. On the other hand, Fu–AuNPs have different MIC values, ranging between 512 and 1024 μg/mL.

The concentration-dependent inhibition of single- and mixed-species biofilms by Fu–AuNPs was discovered. Fu–AuNPs inhibited the biofilms containing *S. aureus*, *S. mutans*, *C. albicans*, and their mixed-species biofilms (*C. albicans* + *S. aureus*/*S. mutans*), most effectively at 512 µg/mL concentration. The maximum inhibitory effect of Fu–AuNPs on the single-species biofilm was found to be closely correlated with the previously reported inhibitory effect on the *P. aeruginosa* biofilm [47]. Fu–AuNPs inhibited biofilms of single microbial species more effectively than polymicrobial biofilms. Previous research has shown that phloroglucinol–chitosan nanoparticles inhibit the mixed biofilm of *C. albicans* + *S. aureus* or *C. albicans* + *S. mutans* at higher concentrations than the individual biofilms of these pathogens [28]. Previous research has shown that a synergistic interaction between *C. albicans* and bacterial pathogens enhances biofilm formation and antimicrobial resistance [53]. 

Furthermore, at concentrations greater than the MIC value of Fu–AuNPs, the effective eradication of established mature biofilms of mono- and mixed-species was observed. Previous research has also found that a higher concentration of AuNPs is needed to eradicate mature biofilms [54]. The higher concentration (> MIC value) required for mature biofilm eradication has been attributed to the recalcitrant nature of the established mature biofilm by the presence of a thick biofilm matrix, which acts as a barrier to drug entry [55]. Although the mechanism of the initial stage of eradication of mature biofilms by Fu–AuNPs cannot be elucidated at this time, the mechanism involves inhibiting biofilm-associated gene expression, altering membrane permeability, and producing reactive oxygen species that can interfere with cellular function, as previously described [56,57]. Because microbial pathogens such as *S. aureus* and *C. albicans* form polymicrobial infections in cystic fibrosis, and *S. mutans* and *C. albicans* form polymicrobial infections in oral diseases [58], the antimicrobial and antibiofilm efficacy of Fu–AuNPs must be studied using synthetic cystic fibrosis and oral saliva to mimic the host environment.

## 4. Materials and Methods

### 4.1. Microbial Strains, Growth Media, and Chemicals

Microbial pathogens, including *Staphylococcus aureus* (KCTC 1916), *Streptococcus mutans* (KCCM 40105), and *Candida albicans* (KCCM 11282), were used in this study [28]. For growing and cultivating the bacterial pathogens *S. aureus* and *S. mutans,* the growth media comprised tryptic soy broth (TSB) and brain heart infusion (BHI) broth (Difco Laboratory Inc., Detroit, MI, USA). However, *C. albicans* was grown in potato dextrose broth (PDB) containing 5% glucose. The visibility and purity of the bacterial colonies structure and *C. albicans* were checked on the agar plate, prepared from the respective broth media by adding bacto agar. The temperature used for the growth of the bacteria and *C. albicans* was 37 °C. Chemical reagents such as gold (III) chloride trihydrate and fucoidan (CAS number 9072-19-9 with a purity of ≥95%) were acquired from Sigma-Aldrich Co. (St. Louis, MO, USA). 

### 4.2. Synthesis of Fucoidan–Gold Nanoparticles

The entire procedure employed in synthesizing the fucoidan-gold nanoparticles (Fu–AuNPs) was adapted from the previous reports, with a slight modification [29] (Figure 1). The reaction was started by dissolving 1 mM HAuCl_4_∙3H_2_O into the sterile deionized water (200 mL) at 60 °C, followed by a continuous stirring. The solution’s alkaline pH (8.5) was adjusted using 0.1 M NaOH. After 1 h stirring, the solution of fucoidan (5 mg) prepared in deionized water was added dropwise, and the pH of the final solution was adjusted to 8.5–9.0. The formation of the AuNPs from the reaction mixture was carried out by striving continuously for 2 h at 60 °C. The formation of the AuNPs was tracked and monitored from the start of the reaction, by measuring the solution’s UV-visible absorption spectra. The scanning of absorption spectra from 200 to 700 nm was carried out using a microplate reader (BioTek, Winooski, VT, USA). In addition, the visible appearance of a wine-red color was also used as an indication of AuNP synthesis. After verifying the synthesis of the AuNPs by measuring the absorption spectra and appearance of the wine-red color, the solution was frozen at −70 °C. The frozen sample was dried in powder form using a freeze drier (FD8518, ilShinBiobase Co. Ltd., Yangju-si, Republic of Korea).

### 4.3. Characterization of Fu–AuNPs

The physiochemical characterization of the Fu–AuNPs was carried out using different instruments, as reported earlier [29]. To determine the shape of Fu–AuNPs, map their elements, and measure the SAED (selected area electron diffraction), field emission transmission electron microscopy (FETEM; JEM-F200, JEOL, Tokyo, Japan) was used. A Fourier transform infrared spectrometer (FTIR, JASCO (FT-4100), Tokyo, Japan) was used to identify the characteristics of functional groups in Fu–AuNPs, with a wavenumber ranging from 400 to 4000 cm^−1^. A Litesizer 500 (Anton Paar, GmbH, Graz, Austria) was used for the size and zeta potential determination of Fu–AuNPs. An X-ray diffractometer (XRD; X-Ray Diffractometer, Rigaku (Japan), Ultima IV) was used to analyze the crystalline nature of the AuNPs. 

### 4.4. Minimum Inhibitory Concentration (MIC) of Fu–AuNPs 

The antimicrobial activity of the of Fu–AuNPs was checked by determining their minimal inhibitory concentration (MIC) towards *S. aureus*, *S. mutans*, and *C. albicans,* using the microbroth dilution technique following the Clinical and Laboratory Standards Institute (CLSI), 2016 [59]. The seed culture of each microbe prepared in their respective growth media was diluted to make the final OD_600_ ~0.05. The 96-well microtiter plate was filled with the diluted cell culture. Concentrations of the Fu–AuNPs ranging between 2048 µg/mL and 64 µg/mL were added to the cell culture in successive wells and incubated at 37 °C for 24 h. The antimicrobial effect of Fu–AuNPs was checked by measuring the OD_600_ using the microplate reader. The concentration of NPs at which more than 90% cell growth was inhibited was contemplated as the MIC. Similarly, the minimum bactericidal or fungicidal concentration (MBC or MFC) of Fu–AuNPs towards bacteria and *C. albicans* was determined in the same way as previously documented [60]. Briefly, MBC was determined by incubating the cells (OD_600_ ~0.05) with the Fu–AuNPs at the MIC concentration and higher MIC value. The incubated cell culture (100 µL) was directly spread-plated on TSA (for bacteria) and PDA (for *C. albicans*). The value of MBC or MFC was decided when there were no visible colonies on the tested concentration of Fu–AuNPs. In addition, using MBC or MFC/MIC ratio, the bactericidal or fungicidal effects of Fu–AuNPs against bacterial species and *C. albicans* were determined, as previously explained [61]. The experiments were implemented in a triad.

### 4.5. Biofilm Assays

The enumeration of the bacterial cells during the biofilm formation, which was treated with different concentrations of Fu–AuNPs, has been carried out by determining the colony-forming units (CFU), as explained earlier [28]. Briefly, the overnight-grown seed cultures of each microbial cell prepared in their respective growth media were standardized and adjusted in such a way as to achieve a final OD_600_ value of 0.05 for bacteria, as well as *C. albicans* cells. These standardized cell cultures were each put into a 96-well microtiter plate with decreasing Fu-AuNP concentrations (from 512 to 128 μg/mL) for the single-species biofilm experiments. The mixed-species biofilm assay was carried out by mixing an equal volume of the bacterial cells (OD_600_~0.05) and *C. albicans* (OD_600_ ~0.05). A similar process was repeated for the dual-species cell culture at decreasing concentrations of Fu–AuNPs. After 24 h incubation, floating cells (planktonic) were removed from each well, followed by three times sterile washing of fixed cells by their respective growth media. New growth media (300 µL) were added to each well, and the surface-adhered cells were scraped with a sterile pipette tip. The obtained cell suspension was serially diluted (10^−8^ dilutions) in a titer plate containing pristine broth, followed by spreading 100 µL each on agar plates (TSA + 2.5 µg/mL of fluconazole for bacteria and PDA + ciprofloxacin 2 µg/mL for *C. albicans*). The 37 °C incubation continued for 24 h for *S. aureus*, while for *S. mutans* and *C. albicans* it extended to 48 h. The colonies grown on the agar surface were totaled, and the CFU values were calculated.

### 4.6. Eradication of Established Mature Biofilms

The disruption efficiency of the Fu–AuNPs towards mature biofilm of solo and conjugate of species was indicated by previous reports [28]. The mature biofilm of single or mixed-species microbial cells was grown in the same way as performed for biofilm assays. The formation of the mature biofilm of single, as well as mixed, microbes was carried out by incubating the cell culture in a 96-well microtiter plate for 24 h, in the same manner as for the biofilm assay, except for the addition of Fu–AuNPs. The plates were freed from planktonic cells, and the biofilm cells were flushed with sterile broth media. Each well was filled with fresh growth media along with different concentrations (256 to 2048 µg/mL) of Fu–AuNPs. Further incubation of the microtiter plate was carried out at 37 °C for 24 h. The planktonic cell culture was removed, and the biofilm cells were washed using sterile broth media. With the help of a pipette tip, the attached cells were scraped out to create a cell suspension in the growth media. A serial dilution (10^−8^) was carried out in the growth media and spread (100 µL)-plated on the agar plates. The colonies’ numbers were used to calculate the CFU values. The experiment was carried out in a group of three and repeated twice.

### 4.7. Examination of Microbial Biofilm Architecture 

The biofilm architecture of singular and combined microbial species was visualized under the impact of NPs using scanning electron microscopy (SEM) [28]. The sample preparation was carried out following the previously reported procedure. Briefly, the nylon membrane of 0.5 × 0.5 cm dimension was placed in each cell of a 24-well microplate. The amount of cell culture, as described in the biofilm assay, either for single or combined species, was placed on the surface of the membrane. Furthermore, these cells were also treated with Fu–AuNPs, and the control group was untreated. The plate was incubated at 37 °C for 24 h. Direct fixing of the biofilm cells using formaldehyde (2%) and glutaraldehyde (2.5%) was performed in the well for 12 h at 4 °C. The unattached cells were removed, followed by three times washing with PBS (7.4). These attached cells were also exposed to increasing ethanol concentrations to remove any remaining moisture. Furthermore, the membranes were freeze-dried (FD8518, ilShinBiobase Co. Ltd., Yangju-si, Republic of Korea) and affixed on the SEM stub. The coating of the cells was carried out by an ion sputterer (E-1010, Hitachi, Japan) for 120 s. The visualization of cells was carried out with the help of the TESCAN microscope (Vega II LSU, Brno, Czech Republic), which was analyzed at 10 kV voltage and with a magnification of 3.0 or 3.5 kx (10 µm or 20 µm). 

### 4.8. Statistical Analysis

With the help of GraphPad Prism 7.0 (GraphPad Software Inc., San Diego, CA, USA), all the graphs were plotted. The one-way ANOVA was used for statistical analysis of each experimental datapoint, and Dunnett’s multiple comparisons tests were also performed. *** *p* < 0.0001, ** *p* < 0.01 and * *p* < 0.05 were considered as significant.

## 5. Conclusions

In the current study, nanotechnology was used as an alternative approach to combat mixed biofilms of *C. albicans* + *S. aureus*/*S. mutans*. Fucoidan, a marine-derived product, was used as a reducing material in the synthesis of gold nanoparticles (Fu–AuNPs). The appearance of the wine-red color of the reaction mixture provided preliminary confirmation. The validation was confirmed once more by in situ monitoring of the specific absorption spectra (570 nm). The results of the detailed instrumental characterization show that the synthesized Fu–AuNPs were both spherical and non-spherical in shape. The high zeta potential value (−36.09 ± 0.63 mV) of the synthesized Fu–AuNPs indicated that they were highly stable. The antimicrobial efficacy, as determined by the MBC or MFC/MIC ratio, was found to be significantly effective against *S. aureus*, *S. mutans*, and *C. albicans*. Fu–AuNPs inhibited initial stage biofilm formation in mono- and polymicrobial species biofilms of *S. aureus/S. mutans* with *C. albicans* in a concentration-dependent manner. The inhibitory effect was also confirmed by examining the biofilm architecture of mono and polymicrobial biofilms, treated with Fu–AuNPs, using SEM analysis. The established mature biofilms of mono- and polymicrobial species were also found to be significantly dispersed by Fu–AuNPs, and the efficacy was found to be higher at higher MIC values. Fu–AuNPs had slightly stronger biofilm inhibitory effects and more mature biofilm eradication against single microbial species than against polymicrobial biofilms. Since fucoidan-AuNPs have never been reported to inhibit a mixed biofilm of bacterial pathogens and *C. albicans* earlier, this highlights the novelty of the current study.

The current research will aid in the control of polymicrobial infections occurring by bacterial and fungal pathogens, through the use of green-synthesized nanoparticles. Fu–AuNPs can be used in the future to create a synergistic effect against polymicrobial biofilms by combining them with antibiotics or antifungals. Future research should look into how Fu–AuNPs interact with polymicrobial planktonic and biofilm cells in a host-mimicking environment, in order to mimic similar effects in the host. The mechanism of action of Fu–AuNPs in inhibiting the initial stage biofilm and eliminating the established mature biofilm at various concentrations must also be investigated.

## Figures and Tables

**Table 1:** MIC and MBC or MFC values of Fu–AuNPs against *S. aureus*, *S. mutans*, and *C. albicans*.

Microbial Pathogens	Antimicrobial Activities		
	MIC (µg/mL)	MBC or MFC (µg/mL)	MBC or MFC/MIC Ratio
*S. aureus*	1024	2048	2
*S. mutans*	>512	1024	2
*C. albicans*	1024	2048	2

## Data Availability

Not applicable.
